# Konstanzer Modellprojekt für psychisch belastete Geflüchtete: Koordinierte psychotherapeutische Behandlung unter Einbezug von Gesundheitspat:innen (KOBEG)

**DOI:** 10.1007/s00115-023-01524-1

**Published:** 2023-08-11

**Authors:** Lea Bogatzki, Julia Miredin, Sophie Millet, Leonie Lipinski, Madlen Molle, Brigitte Rockstroh, Daniela Mier, Michael Odenwald

**Affiliations:** 1 Universität Konstanz, Konstanz, Deutschland; 2 vivo international e. V., Konstanz, Deutschland

**Keywords:** Geflüchtete, Peers, Psychische Gesundheit, Psychotherapie, Gesundheitssystem, Refugees, Peers, Mental health, Psychotherapy, Healthcare system

## Abstract

Geflüchtete mit psychischen Störungen sind mit Zugangsbarrieren zum psychiatrisch-psychotherapeutische Versorgungssystem konfrontiert. Um diesen Barrieren zu begegnen, wurde im Landkreis Konstanz ein Modellprojekt zur Unterstützung der Gesundheitsversorgung und Integration psychisch belasteter Geflüchteter etabliert (Koordinierte psychotherapeutische Behandlung unter Einbezug von Gesundheitspat:innen; KOBEG) und in einer ersten 3‑jährigen Modellphase evaluiert. Über eine zentrale Koordinierungsstelle werden die Geflüchteten an lokale, niedergelassene Psychotherapeut:innen vermittelt. Sprach- und kultursensitiv geschulte Gesundheitspat:innen (Peers) betreuen und begleiten die Geflüchteten während ihrer Therapie. Die Inanspruchnahme des vermittelten Therapieangebots und Erfahrungen beteiligter Therapeut:innen dienen als Indikatoren für die Effektivität der patengestützten Therapievermittlung. Die Wirksamkeit der therapeutischen Maßnahmen und Begleitung durch Gesundheitspat:innen wurde anhand der psychischen Symptombelastung (Symptomchecklist-27) und Funktionsbeeinträchtigung (Work and Social Adjustment Scale) vor und nach durchschnittlich 10 Monaten Projektteilnahme evaluiert. Im Zentrum der Untersuchung stehen die Geflüchteten (Angebotsinanspruchnahme und psychische Belastung) sowie die Psychotherapeut:innen (berichtete Erfahrungen). Erste Ergebnisse belegen eine überdurchschnittlich hohe Belastung der Geflüchteten zum Zeitpunkt des Erstgesprächs, eine hohe Teilnahmequote und eine geringe Abbruchrate. Die Verlaufserhebung zeigt signifikante Verbesserungen hinsichtlich der psychischen Symptombelastung sowie Funktionsbeeinträchtigung. Die (qualitative) Befragung der Psychotherapeut:innen unterstreicht die Effektivität von KOBEG, 93 % der Therapeut:innen beabsichtigen im Rahmen des Projekts weiterhin mit Geflüchteten zu arbeiten.

**Zusatzmaterial online:**

Die Onlineversion dieses Beitrags (10.1007/s00115-023-01524-1) enthält zusätzliches Material. Beitrag und Zusatzmaterial stehen Ihnen auf www.springermedizin.de zur Verfügung.

## Abstract (en)

Refugees with mental disorders are confronted with access barriers to the psychiatric and psychotherapeutic care system. In order to counter these barriers, a model project to support the health care and integration of mentally distressed refugees was established in the district of Konstanz (coordinated psychotherapeutic treatment involving trained peer support; KOBEG) and evaluated in an initial 3‑year model phase. A coordination center refers the patients to local certified psychotherapists. Language and culturally sensitive trained mentors (peers) support and accompany the refugees during the therapy. The utilization of the refered therapy and the experiences of the participating therapists serve as indicators for the effectiveness of the peer-supported therapy referral. The effectiveness of therapeutic measures and support by trained peers was evaluated on the basis of the psychological symptom burden (symptom checklist 27) and functional impairment (work and social adjustment scale) before and after an average of 10 months of project participation. The study focuses on the refugees (use of services and mental distress) and the psychotherapists (reported experiences). Initial results showed an above-average stress level among the refugees at the time of the initial interview, a high participation rate and a low drop-out rate. The follow-up survey showed significant improvements in terms of psychological symptom burden as well as functional impairments. The (qualitative) survey among psychotherapists underlines the effectiveness of KOBEG, 93% of the therapists intend to continue working with refugees within the framework of the project.

Weltweit sind aktuell über 100 Mio. Menschen auf der Flucht [29]. Strukturelle Barrieren erschweren dieser besonders vulnerablen Zielgruppe den Zugang zum krankenkassenfinanzierten Regelversorgungssystem [7]. Das nachfolgend vorgestellte Modellprojekt wurde 2017 für den Landkreis Konstanz entwickelt, um mittels koordinierter psychotherapeutischer Behandlung unter Einbezug von Gesundheitspat:innen (KOBEG) den Zugang zur Regelversorgung für belastete Geflüchtete zu verbessern. Die nachfolgend berichteten Evaluationsergebnisse dokumentieren Notwendigkeit, Machbarkeit und erste Hinweise auf die Effektivität des Modellprojekts.

## Hintergrund

Unfreiwillige Migration aufgrund von Flucht vor Krieg und Verfolgung geht mit einem hohen psychischen Morbiditätsrisiko einher [7]. Neuere Studien berichten hohe Prävalenzen für psychische Störungen wie Depressionen (40,9 % [19]; 31,5 % [5]) und Posttraumatische Belastungsstörungen (PTBS: 43,0 % [19]; 31,46 % [5]). In einer von der Bundesregierung in Auftrag gegebenen Studie, schätzten Expert:innen, dass 50 % der in den letzten Jahren nach Deutschland gekommenen Geflüchteten traumabedingte psychische Störungen haben und etwa 25 % von ihnen professioneller psychotherapeutischer Hilfe bedürfen [17].

Das Recht auf gesundheitliche Grundversorgung ist auf internationaler Ebene für Geflüchtete geschützt [3]. Bis heute besteht dennoch eine große Diskrepanz zwischen Bedarf und praktischer Versorgung. Auf Grundlage des Asylbewerberleistungsgesetztes (AsylbLG) werden Gesundheitsleistungen nur eingeschränkt gewährt; insbesondere Psychotherapie wird häufig abgelehnt (je nach Kostenträger Ablehnungsquote bis zu 41 % [3]). Sprachmittlungkosten werden nicht von den Krankenkassen übernommen. Nach 18 Monaten erhalten Geflüchtete eine elektronische Gesundheitskarte und sog. Analogleistungen, Kostenträger ist im Rahmen des AsylbLG jedoch weiterhin das Sozialamt. Die Bundespsychotherapeutenkammer betrachtet Geflüchtete daher als eine systemisch diskriminierte Gruppe bez. des Zugangs zu Psychotherapie [8]. Über spezialisierte Einrichtungen (Psychosoziale Zentren der Bundesarbeitsgemeinschaft für Flüchtlinge und Folteropfer, BafF) kann nur ein kleiner Anteil der Zielgruppe versorgt werden und es bestehen lange Wartezeiten [3]. Weitere Zugangsbarrieren zum Versorgungssystem sind Wissensdefizite (über psychische Störungen und deren Behandlung) und administrative Hürden für Psychotherapeut:innen [11, 23]; diese erschweren Geflüchteten den Zugang zum Versorgungssystem und erklären die verhältnismäßig geringe Inanspruchnahme von Psychotherapie [4, 23].

### Modellprojekt

Mit dem Ziel, psychisch belasteten Geflüchteten die Angebote der Regelversorgung zugänglich zu machen, entstand 2017 das Modellprojekt KOBEG an der Universität Konstanz in enger Zusammenarbeit mit dem gemeinnützigen Verein vivo international e. V.

Das Modellprojekt im Landkreis Konstanz beinhaltet die Elemente einer koordinierten Versorgung sowie den Einsatzes von Peers (Gesundheitspat:innen).

### Fragestellungen der Untersuchung

In dieser Evaluierung des Modellprojekts werden Notwendigkeit (anhand der Symptomschwere), Effektivität des Projekts (Inanspruchnahme durch Patient:innen; Beurteilung durch Psychotherapeut:innen) und erste Hinweise auf die Wirksamkeit der therapeutischen Maßnahmen und Begleitung durch Gesundheitspat:innen (Veränderung der Symptomatik und Funktionsbeeinträchtigung) untersucht.

## Methoden

### Koordinierte psychotherapeutische Behandlung unter Einbezug von Gesundheitspat:innen (KOBEG)

Basierend auf langjähriger Erfahrung in der Diagnostik und Therapie von Geflüchteten [1] wurde im Landkreis Konstanz eine zentrale Koordinationsstelle eingerichtet und die umfangreiche Vernetzung mit bestehenden Strukturen der Flüchtlingshilfe ausgebaut. Parallel wurden vermehrt Personen als Gesundheitspat:innen oder Sprachmittler:innen rekrutiert und ausgebildet; diese sollten Muttersprachler:innen eines der Hauptherkunftsländer sein (Afghanistan, Iran und Syrien), solide Deutschkenntnis (mindestens B2), eigenen Flucht- oder Migrationshintergrund sowie psychische Stabilität aufweisen. Alle Gesundheitpat:innen wurden in einem Bewerbungsverfahren ausgewählt, durchliefen spezifische Schulungen (6 Termine à 3 h) zum deutschen Gesundheitssystem, psychischen Störungen insbesondere PTBS, Selbstfürsorge und Abgrenzung, Umgang mit Krisen, Sprachmittlung sowie zur Fragebogenerhebung mit Patient:innen, wurden von Psycholog:innen angeleitet und waren verpflichtet an Supervisionssitzungen teilzunehmen. Geflüchtete mit psychischer Belastung wurden von kooperierenden Institutionen im Landkreis angemeldet und in die Koordinationsstelle eingeladen. Dabei standen die Aufklärung, Entscheidung bez. Projekteinschluss, Datenerhebung mit dem strukturierten diagnostischen Interview MINI [24] und Fragebögen (SCL-27 [10], WSAS [16]), schriftliche Einwilligung und Vermittlung von Information im Vordergrund. Bei Projekteinschluss wurden Betroffene an kooperierende ambulante Psychotherapeut:innen im Landkreis vermittelt. Praxen in der Versorgungsstruktur wurden durch Informationsveranstaltungen, kostenfreie Fortbildungsangebote (bspw. Arbeit mit Sprachmittlung in der Psychotherapie) und durch persönliche Kontaktaufnahme gewonnen. Etwa 25 Psychotherapeut:innen waren zum Zeitpunkt der Studie beteiligt, zudem bestand eine enge Kooperation mit dem lokalen Ausbildungsinstitut und mit der Hochschulambulanz des Lehrstuhls Klinische Psychologie und Psychotherapie. Gesundheitspat:innen überbrückten durch regelmäßige Kontakte die Wartezeit und unterstützten durch aufsuchende, individuell an die Bedürfnisse der Patient:innen angepasste Begleitung den Übergang in die Behandlung. Gesundheitspat:innen leisteten dabei emotionale Unterstützung, halfen bei der Organisation von Terminen, begleiteten zu ärztlichen und oder behördlichen Terminen im Rahmen der Behandlung (z. B. zur Erlangung von Behandlungsscheinen), vermittelten kulturelles Wissen (bspw. „Was ist Psychotherapie?“), motivierten zur fortgesetzten Wahrnehmung der Therapietermine und fungierten als Kulturmittler:innen. Bei Sprachmittlungsbedarf in der Therapie kamen Sprachmittler:innen zum Einsatz. Die konzeptionelle Abgrenzung von Sprachmittelnden und Gesundheitspat:innen ist hierbei wichtig: Sprachmittelnde erscheinen nur zu den Therapieterminen und haben keinen weiteren Kontakt zu den Patient:innen; Gesundheitspat:innen betreuen und begleiten Patient:innen im persönlichen Kontakt. In unserer Pilotstudie konnten Gesundheitspat:innen jedoch bei einer geflüchteten Person beide Rollen einnehmen.

Das Unterstützungsangebot der Koordinationsstelle an Psychotherapeut:innen umfasste kostenlose Fortbildungen (z. B. Psychotherapie mit Sprachmittlung, narrative Expositionstherapie bei PTBS), Intervisionsgruppen sowie administrative Unterstützung (Organisation und Abrechnung von Sprachmittlung, Unterstützung bei Kostenbeantragung, Beratung bei Fragen zum Asylverfahren etc.). Die kostenlosen Fortbildungen wurden hierbei hochfrenquentiert genutzt und waren bei jedem Durchgang ausgebucht. Die administrative Unterstützung erfolgte primär hinsichtlich der Abrechnungsmodalitäten, bei Fragen zu Gutachten im Asylprozess oder zur Arbeit und Kooperation mit den Sprachmittelnden. Das akkreditierte Intervisionsangebot wurde von ca. einem Drittel der Therapeut:innen regelmäßig, von den anderen eher sporadisch genutzt; häufige Themen waren dabei die Herausforderung der Arbeit mit Sprachmittlung und spezifische Belastungen (bspw. Umgang mit herkunftslandspezifischen Stressoren und Besonderheiten im Asylprozess, z. B. wenn Atteste verfasst werden sollten). Die beteiligten Therapeut:innen waren neben zwei Tiefenpsycholog:innen und einem Analytiker überwiegend verhaltenstherapeutisch orientiert.

Gesundheitspat:innen und Sprachmittler:innen erhielten regelmäßige Supervisionsangebote. Abgrenzung, eigene Betroffenheit, Umgang mit den Gefühlen Wut und Scham, Selbstfürsorge, Kultursensibilität, Verantwortungsübernahme und Abgabe im Projekt stellten häufige Themen der Supervision dar.

Die eingeschlossenen Patient:innen wurden im Abstand von 4 Monaten durch die Koordinierungsstelle zu Katamnesegesprächen eingeladen, unabhängig vom Erfolg der Vermittlungsversuche in die Regelversorgung.

### Studienplan der Projektevaluierung

Im Zentrum der Untersuchung stehen die psychisch belasteten Geflüchteten (Inanspruchnahme Angebot und psychische Belastung) sowie die Psychotherapeut:innen (berichtete Erfahrungen). Einschlusskriterien der Geflüchteten waren die Indikation für eine ambulante Psychotherapie (z. B. Vorliegen einer psychischen Störung, anhand des MINI [24]) sowie die Bereitschaft zur Beteiligung an den Projektmaßnahmen. Ausschlusskriterien für eine Projektteilnahme war das Vorliegen einer psychischen Störung, die eine stationäre Therapie erforderte (z. B. akute Psychose, akute Suchtproblematik, akute Suizidalität anhand des MINI). In einem unkontrollierten Längsschnittdesign wurde der Symptomverlauf wiederholt erfasst. In dieser Studie werden Daten aus dem Erstgespräch und der letzten, routinemäßig erfassten Verlaufsmessung verglichen. In die Studie aufgenommen wurden alle Patient:innen, deren letzte Verlaufsmessung mindestens 4 Monate nach dem Erstgespräch stattgefunden hat, da so angenommen werden konnte, dass neben der Gesundheitspat:innen-Betreuung auch die Psychotherapie bereits begonnen hatte (Wartezeiten auf einen Therapieplatz durchschnittlich 15,5 Wochen [18]). Die Erhebungen fanden zwischen 2017 und 2020 statt, Fragebögen und Daten zur Anzahl der Therapietermine wurden im persönlichen Kontakt mit professioneller Sprachmittlung erhoben. Alle Fragebögen wurden im direkten Kontakt mit den Patient:innen und mithilfe ausgebildeter Sprachmittler:innen vom Deutschen in die jeweilige Muttersprache übersetzt. Die Erstgespräche wurden von Psycholog:innen des Projektteams durchgeführt, die Follow-ups (FUs) von trainierten Psychologiestudierenden. Im Zuge der Corona-Pandemie (ab April 2020) wurden die Gesundheitspat:innen zum selbstständigen Durchführen der FU-Fragebögen ausgebildet.

In einer anonymen Onlinebefragung über das Online-Tool Unipark, https://www.unipark.com, Tivian XI GmbH, Köln, Deutschland, wurden Projekttherapeut:innen, die zwischen 2017 und 2018 am Modellprojekt beteiligt waren, zu ihren Erfahrungen bei den Behandlungen der Geflüchteten befragt.

### Stichprobe

Insgesamt wurden 51 Geflüchtete (43 Männer und 8 Frauen, Alter bei Erstgespräch M = 25,69 Jahre, SD = 7,58, Range 15–43) in die Studie aufgenommen. Die häufigsten Herkunftsländer waren Afghanistan (33 %), Iran (22 %), Syrien (16 %) und Irak (12 %). Zum Zeitpunkt des Erstgesprächs lebten 67 % in einer Flüchtlingsunterkunft, die überwiegende Mehrheit war 2015 (41 %) und 2016 (24 %) nach Deutschland eingereist. 33 % befanden sich bei Projektvorstellung im Erst-, 12 % weitere im Folgeverfahren, 29 % besaßen eine Duldung bzw. vorübergehende Aussetzung der Abschiebung, 22 % hatten eine Aufenthaltsgenehmigung, bei 4 % lagen keine Angaben vor. Insgesamt liegen bei 39 Geflüchteten FU-Daten vor, Gründe für Dropout waren „keine Projektteilnahme“ (*n* = 6) und „Beendigung der Projektteilnahme vor Durchführung eines FU“ (*n* = 3); für 3 weitere Personen fehlen FU-Daten vollständig. Von den *n* = 39 Personen der Evaluation waren 32 Männer und 7 Frauen im Alter zwischen 16 und 43 Jahren (M = 26,23, SD = 7,93). Die FU fanden im Mittel 10,08 Monate nach dem Erstgespräch statt (SD = 4,33, Rang = 4–21, Anzahl FUs Range = 1–3).

Voraussetzung für die Teilnahme an der Therapeut:innen-Befragung war mindestens eine abgeschlossene Therapie im Rahmen des Projektes. 14 Therapeut:innen nahmen daran teil (2 männlich; 9 approbiert, 5 in Ausbildung), von denen 11 als Schwerpunktverfahren Verhaltenstherapie, 2 Tiefenpsychologie und 1 Psychoanalyse angaben. Die Befragung umfasste die Erfahrung von 19 innerhalb des Projekts durchgeführten Therapien.

### Instrumente und Indizes

Zur Einschätzung der psychischen Beschwerden kam die *Symptomchecklist-27* (SCL 27; [10]), ein Verfahren zur Selbstbeurteilung der subjektiv empfundenen Belastung durch körperliche und psychische Symptome innerhalb der letzten 7 Tage, zum Einsatz. Sie umfasst 27 Items und setzt sich aus 6 Subskalen zusammen. Validität und Reliabilität des Instruments wurden bestätigt, Normwerte des Instrumentes liegen aus mehreren Populationen vor [10]. Die erlebte Einschränkung der Leistungsfähigkeit und Alltagsfunktionalität wurde mittels der *Work and Social Adjustment Scale* (WSAS) erhoben, bestehend aus 5 Items und einer 8‑stufigen Skala. Die WSAS gilt als verlässliches, valides unidimensionales Messinstrument für den Schweregrad funktioneller Beeinträchtigungen [16]. Zum besseren Verständnis wurde für SCL 27 und WSAS eine visuelle Ratingskala entworfen.

Therapeut:innen-Befragung: Ein Onlinefragebogen erfasste die Erfahrungen der Therapeut:innen bezüglich Therapieerfolg, Sprachmittlung, Gesundheitspat:innen, Koordinationsstelle sowie Belastungen und Bereicherungen. Alle Themenkomplexe wurden in Ratingskalen abgefragt, zusätzlich gab es zu jedem Themenbereich die Kategorie „Beurteilung in eigenen Worten“, die hier qualitativ analysiert wird.

### Statistische Auswertung

Die *Notwendigkeit* der Behandlung psychisch belasteter Geflüchteter wurde anhand des Vergleichs der Symptombelastung der aktuellen Stichprobe (*n* = 39) mit einer Normstichprobe von Psychotherapiepatient:innen mit affektiver Erkrankung (*n* = 2717, [20]) untersucht (One-Sample t‑Tests; Cohen’s d).

Die *Effektivität *wurde aus der Inanspruchnahme bzw. Abbruch des Projektangebots von Studienaufnahme bis zum Zeitpunkt des FU und den qualitativen Aussagen der teilnehmenden Psychotherapeut:innen abgeleitet. Die Daten zur Bewertung der Effektivität wurden sowohl an Geflüchteten als auch an Therapeut:innen erhoben. Vergleichsmaßstab war die Therapieabbruchrate (nach Probatorik) an deutschen Hochschulambulanzen von 14,1 % ([9]; χ^2^-Test). Die qualitative Auswertung der Aussagen der Psychotherapeut:innen zu ihren Erfahrungen bei Behandlungen im Modellprojekt erfolgte anhand Mayrings qualitativer Inhaltsanalyse [15]. Aufgrund der explorativen Natur dieser Untersuchung werden die Daten induktiv mittels zusammenfassender Inhaltsanalyse ausgewertet. Die Aussagen der Therapeut:innen werden dabei zusammengefasst und zu Kategorien zugeteilt, die sich induktiv aus dem Datenmaterial ergeben.

Die Wirksamkeit der therapeutischen Maßnahmen und Begleitung durch Gesundheitspat:innen wurde an der Reduktion der Symptombelastung und Funktionsbeeinträchtigung vom Erstgespräch zum FU mittels t‑Tests für abhängige Stichproben gemessen. Studienobjekt sind hierbei die Geflüchteten. Zudem wurde mittels einfaktorieller ANCOVA mit Messwiederholung überprüft, ob die Veränderung der Gesamtwerte von den Variablen Alter, Geschlecht, Dauer der Projektteilnahme (zeitlicher Abstand zwischen Erstgespräch und FU) und Anzahl an Therapiestunden beeinflusst wurde. Die Effektstärke wurde mit dem partiellen η^2^ angegeben.

## Ergebnisse

### Notwendigkeit/Bedarf

**Figure 1:**
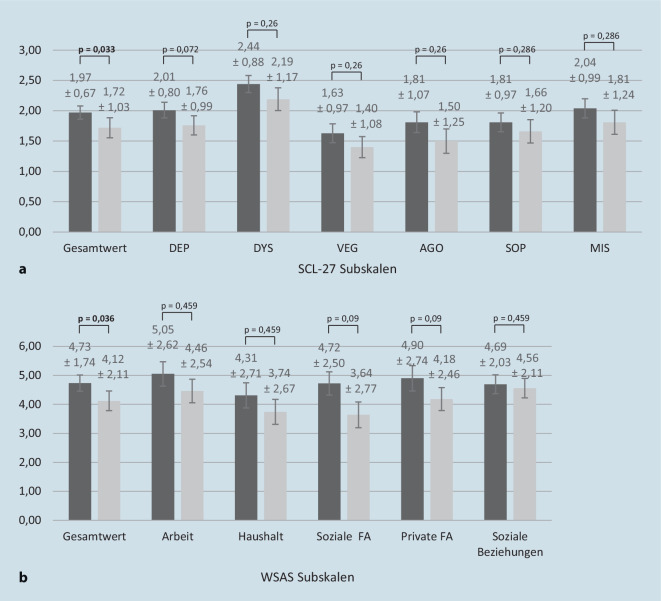

Zum Zeitpunkt des Erstgesprächs zeigt sich bei den Geflüchteten im SCL-27 (Abb. 1) eine signifikant höhere mittlere Gesamtbelastung (GSI) als in der klinischen Normstichprobe (t(50) = 7,51, *p* < 0,001, d = 1,05). Dies zeigt sich auch in Bezug auf alle Subskalen der SCL-27 (alle *p* ≤ 0,002).

### Effektivität

#### Inanspruchnahme der Projektunterstützung

Die Inanspruchnahme war hoch. Nur 17,6 % (*n* = 9) hatten die angebotene Unterstützung durch das Projekt sofort oder kurz nach dem Erstgespräch abgelehnt, sodass keine Vermittlung in Regelpsychotherapie stattfand.

#### Therapieabbruch

Von den längsschnittlich untersuchten 39 Geflüchteten, die in Psychotherapie vermittelt wurden, hatten bei der FU-Untersuchung 11 Personen ihre Psychotherapie bereits regulär beendet, 21 befanden sich noch in einer laufenden Psychotherapie. Vier Geflüchtete hatten die Therapie abgebrochen, eine zusätzliche Therapie war durch eine Therapeutin abgebrochen worden. Zwei warteten auf den Beginn ihrer Therapie. Zum Zeitpunkt des FU hatten die Geflüchteten zwischen 0 und 80 Therapietermine wahrgenommen (M = 15,64, SD = 15,93). Bei 3 Geflüchteten fehlen Werte zur konkreten Stundenanzahl. Daraus ergibt sich eine Therapieabbruchrate von 13,5 % (*n* = 5/37; χ^2^ = 0,1351, *p* = 0,713208).

#### Erfahrung der Psychotherapeut:innen

Insgesamt lässt sich eine positive Einschätzung hinsichtlich Effektivität, insbesondere in Zusammenarbeit mit den Gesundheitspat:innen sowie mit der Koordinationsstelle im freien Antwortverhalten festhalten (s. Anhang A). Eine Therapeutin berichtete: „Alpträume, Schlafstörungen und Schwere der Depression haben sich gebessert. Einen Raum zu haben, in dem er sein Grauen berichten kann und sich entladen kann, ist für ihn wichtig“ (Th5). Als bereichernd wurden neue Einblicke sowie positive Entwicklungen der Patient:innen erlebt. Belastende Aspekte stellten die geringe Therapiemotivation und geringe Eigenverantwortung seitens der Patient:innen, ein höherer Anteil an sozialarbeiterischen Tätigkeiten sowie Berichte der Patient:innen dar. Zeitliche Koordinierung, Zweifel an Übersetzungen sowie dominantes Verhalten der Sprachmittelnden wurden herausfordernd beschrieben, insgesamt wurden die Sprachmittelnden aber hilfreich und kompetent erlebt. Die Unterstützung durch die Gesundheitspat:innen wurde sowohl für Patient:innen als auf für Therapeut:innen als hilfreich erlebt. Bsp. „Der Gesundheitspate war sehr stützend für den Patienten, der sonst die Therapie wahrscheinlich direkt am Anfang abgebrochen hätte“ (Th1). 13 der 14 Therapeut:innen (93 %) konnten sich vorstellen, auch in Zukunft weiter mit Geflüchteten zu arbeiten. Sie betonten dabei die Wichtigkeit und Machbarkeit des Projekts. Beispielaussagen machen dies besonders greifbar: „Durch das Projekt ist jeder und jede Psychotherapeut:in in der Lage, auch den höheren Aufwand etwas abzufedern und sprachliche Barrieren und kulturelle Verständnisprobleme zu überwinden“ (Th7) oder: „Habe vorher ohne Unterstützung versucht, mit Geflüchteten zu arbeiten. War extrem zeitaufwendig und frustrierend, da bei 3 Versuchen keine Therapie zustande gekommen ist. … Im Projekt lief das Ganze sehr viel reibungsloser ohne Riesenmehraufwand“ (Th12).

#### Wirksamkeit der therapeutischen Maßnahmen und Begleitung durch Gesundheitspat:innen

Die 39 Geflüchteten in der Längsschnittstudie berichteten zum Zeitpunkt des FU im Schnitt von einer leicht reduzierten psychischen Gesamtbelastung im Vergleich zum Erstgespräch (kleiner bis mittlerer Effekt; *p* = 0,033; Abb. 1). In den einzelnen SCL-27-Subskalen erreichte die Symptomreduktion kleine oder kleine bis mittlere Effekte (alle *p* ≥ 0,072; Abb. 1). Ferner ergaben sich keine signifikanten Einflüsse der Kovariablen Alter (F[1, 35] = 0,23, *p* = 0,635, η^2^ = 0,01), Geschlecht (F[1, 35] = 0,22, *p* = 0,642, η^2^ = 0,01), Teilnahmedauer am Projekt (F[1, 35] = 0,82, *p* = 0,371, η^2^ = 0,02) und Anzahl an Therapiestunden (F[1, 34] = 0,12 *p* = 0,732, η^2^ < 0,01). In Bezug auf die Veränderung der globalen Funktionalität (WSAS-Gesamtwert) zeigte sich eine signifikante, kleine bis mittlere Verbesserung im Verlauf (*p* =0,036; siehe Abb. 1), während auf Subskalenebene die Veränderungen nicht statistisch bedeutsam waren (alle *p* ≥ 0,09, alle Effekte klein oder klein bis mittel; siehe Abb. 1).

## Diskussion

Die vorliegende Untersuchung zeigt Effektivität und Machbarkeit der Behandlung psychisch belasteter Geflüchteter in Regelversorgungsstrukturen. In Deutschland gibt es unseres Wissens nach bis heute keine vergleichbaren Daten aus einem koordinierten und Peer-unterstützen Versorgungsmodell für Geflüchtete mit psychischen Störungen.

### Notwendigkeit

Die hohe Belastung der Geflüchteten im Vergleich zu einer Normstichprobe von Patient:innen mit affektiver Erkrankung macht den starken Behandlungsbedarf deutlich und deckt sich mit vorherigen Befunden [5, 19].

### Effektivität

Die Inanspruchnahme von Psychotherapie ist mit 82,4 % (*n* = 42) hoch und vergleichbar mit anderen Patient:innen-Populationen [2]. Die hier berichtete Abbruchrate von 13,5 % (*n* = 5, inklusive in der Probatorik) weicht nicht von der nichtgeflüchteter Patient:innen ab (14,1 % nach Probatorik [9]). Unsere Ergebnisse sind eine Momentaufnahme aus der laufenden Versorgung, da nicht alle Therapien bereits abgeschlossen waren und potenziell noch hätten abgebrochen werden können. Da Therapieabbrüche vor allem in der Probatorik und in der ersten Phase der Therapie auftreten [9, 21], ist davon auszugehen, dass sich dieses Bild nicht wesentlich verändern wird. Der Vergleich mit Interventionsstudien bei Geflüchteten mit PTBS erlaubt eine weitere Einordnung der Daten: Effektivität wurde gezeigt [13, 27, 28], jedoch weisen einige Studien sehr hohe Abbruchraten von über 50 % auf (z. B. [25]). Es ist zu beachten, dass diese Studien mit standardisierten, kurzen Interventionen (geplante Sitzungszahl M = 8, Range 2–16; Dauer M = 102 min, Range 60–240 min [13]) außerhalb der kassenfinanzierten Regelversorgung stattfanden. Unsere Studie hingegen unterstützt die Machbarkeit von Behandlungen in Regelversorgungsstrukturen.

Die Befragung der Psychotherapeut:innen unterstreicht die Effektivität des Modellprojekts, wobei insbesondere die Koordinationsstelle und der Einbezug von Gesundheitspat:innen als unterstützend und entlastend erlebt wird. Gleichzeitig zeigen sich bekannte Barrieren und Schwierigkeiten [22]. Das Modellprojekt schafft hier jedoch einen konstruktiven Zugang und Umgang durch Unterstützungsangebote sowie den Einsatz der Gesundheitspat:innen, sodass 93 % der Therapeut:innen (*n* = 13) angeben, dass sie weiterhin mit Geflüchteten im Rahmen des Projekts arbeiten würden.

### Wirksamkeit der therapeutischen Maßnahmen und Begleitung durch Gesundheitspat:innen

Im Schnitt zeigen sich eine reduzierte psychische Gesamtbelastung (SCL-27) im Projektverlauf sowie eine Verbesserung der globalen Funktionalität (WSAS). Diese kleinen Veränderungen sind vor dem Hintergrund von Verlaufsstudien, in denen sich die Symptomatik unbehandelter Geflüchteter mit PTBS nicht verbesserte [6, 12], als Erfolg zu werten. Außerdem lebten viele der Patient:innen zum Zeitpunkt der Therapie noch in Gemeinschaftsunterkünften und befanden sich im Asylverfahren, d. h. die Lebensumstände waren vermutlich von Unsicherheit, Unruhe und Postmigrationsstressoren geprägt [26]. Angesichts dieser destabilisierenden Lebenssituation ist eine initiale Stabilisierungsphase erforderlich, in der oft nicht symptomfokussiert gearbeitet werden kann [14]. Gerade in dieser Phase hat die Zusammenarbeit mit Gesundheitspat:innen eine große Bedeutung, um Therapieabbrüche zu vermeiden. Ein randomisiert-kontrolliertes Studiendesign ist jedoch für die weitere Evaluierung erforderlich.

### Limitationen

Limitationen sind eine kleine und überwiegend männliche Stichprobe sowie ein unkontrolliertes Design, eine pragmatisch kurze Diagnostik und aufgrund von Erreichbarkeit und Motivation der Geflüchteten unregelmäßige Abstände vom Erstgespräch zum FU-Termin. Zusätzlich wurden im Zuge der Corona-Pandemie die Fragebogenerhebung von Psycholog:innen auf Gesundheitspat:innen umgestellt. Durch die verfahrensoffene Konzeption des Projekts sind Psychotherapeut:innen verschiedener Therapieschulen beteiligt, die selbstverantwortlich über das indizierte Vorgehen entscheiden. Dies geht mit einer Heterogenität der psychotherapeutischen Interventionen und in der wissenschaftlichen Evaluierung mit methodischen Problemen (interne Validität) einher. Auch ist in dieser ersten Evaluation der Effekt der Psychotherapie nicht vom Effekt der Gesundheitspat:innen zu trennen. Die Therapeut:innen-Befragung wurde aus praktischen Gründen bereits 2018 durchgeführt, was zu einer Verzerrung der berichteten Ergebnisse führen kann, da viele der Behandlungen deutlich länger anhielten.

### Implikationen und Ausblick

Die hier beschriebene erste Evaluierung von KOBEG ist vielversprechend. Aktuell wird eine randomisiert-kontrollierte Studie zu diesem Ansatz durchgeführt. Bei positiver Evaluierung sollte der Auf- und Ausbau *koordinierter und Peer-unterstützter Versorgungsstrukturen *für Geflüchtete auf der kommunalen Ebene gefördert werden.

## Fazit für Praxis

- Die besondere Schutzbedürftigkeit einer sehr vulnerablen Gruppe im Hinblick auf psychische Belastungen wird in der Studie deutlich.
- Eine *koordinierte* und *Peer-unterstützte Versorgungsstruktur* scheint hilfreich, um eine strukturell benachteiligte Patient:innengruppe wie Geflüchtete mit psychischen Störungen in die Regelversorgung zu integrieren.
- Niedergelassene Psychotherapeut:innen bewerten eine koordinierte und Peer-unterstützte Versorgungsstruktur als hilfreich für die Versorgung von Geflüchteten mit psychischen Störungen.
- Sprachmittler:innen-Pools stellen eine wichtige Voraussetzung für die Durchführung indizierter psychotherapeutischer Behandlungen bei Geflüchteten dar.
- Vernetzung und interdisziplinärer Zusammenarbeit sollen gefördert werden, um die strukturelle Benachteiligung Geflüchteter im Zugang zu Psychotherapie schrittweise zu mildern und langfristig einen diskriminierungsfreien Zugang zum Menschenrecht auf Gesundheit zu ermöglichen.

### Supplementary Information




