# Supplementary material for: The Konstanz model project for refugees with mental disorders: Coordinated psychotherapeutic treatment involving trained peer support (KOBEG)
Source: Nervenarzt. 2023 Aug 11;94(11):1026–33. [Article in German] doi: 10.1007/s00115-023-01524-1 (PMC10620279; doi:10.1007/s00115-023-01524-1)
Supplement: Supplementary file 1 [file 115_2023_1524_MOESM1_ESM.docx]

Anhang A:

| Grundthema und Fragestellung | Qualitative Angaben Psychotherapeut:innen | Themencluster |
| --- | --- | --- |
| 1.Einschätzung Veränderung Symptomatik | N = 14 |  |
|  | 1a. Positive Aspekte | 1aa. Verbesserung der Symptomatik (*n* = 4) 1ab. Schaffung eines Raums für das Sprechen über Erlebtes und Probleme, was viele der Patienten vorher nicht kannten (*n* = 5) 1ac. Entlastung durch Verbesserung der Lebenssituation in Form einer gefundenen Arbeit (Th19). *Bsp. „Alpträume, Schlafstörungen und Schwere der Depression haben sich gebessert. Einen Raum zu haben, in dem er sein Grauen berichten kann und sich entladen kann, ist für ihn wichtig.“* (Th5) |
| 2. Einschätzung Belastung und Bereicherung | N = 10 |  |
|  | 2a. Bereichernde Aspekte | 2aa. Neue Einblicke (*n* = 2)  2ab. Positive Entwicklungen der Patienten (*n* = 2) |
|  | 2b. Belastende Aspekte | 2ba. Verhalten des Patienten  - geringe Therapiemotivation (*n* = 2) - geringe Eigenverantwortung (*n* = 1)  - unklaren Therapienutzen (*n* = 1).  2bb. Belastende Berichte der Patienten (*n* = 6) - die Lebensgeschichten (*n* = 4)  - die Lebenssituation der Patienten in Deutschland (*n* = 2).  2bc. Sozialarbeiterische Tätigkeiten (N = 2) |
| 3. Sprachmittlung | N = 9 |  |
|  | 3a. Positive Aspekte | 3aa. Hilfreiche Unterstützung (*n* = 2)  3ab. Kompetente Arbeit (*n* = 4) |
|  | 3b. Negative Aspekte | Vier Therapeut:innen bewerteten die Arbeit mit der Sprachmittlung als problematisch.  3ba. Zeitliche Koordinierung (*n* = 2), 3bb. Zweifel an der Übersetzung (*n* = 1) 3bc. dominantes Verhalten (*n* = 2)  3bd. Trennung von Inhalt und Gefühl durch die Übersetzung (*n* = 1) gesehen. |
| 4. Gesundheits- patInnen | N = 9 |  |
|  | 4a. Positive Aspekte | 4aa. Unterstützung durch die Gesundheitspat:innen, zum einen für den Patienten (*n* = 2) als auf für den Therapeuten (*n* = 3).  Bsp. „*Der Gesundheitspate war sehr stützend für den Patienten, der sonst die Therapie wahrscheinlich direkt am Anfang abgebrochen hätte“* (Th1).  4ab. Verlässlichkeit der Gesundheitspat:innen (n = 2) |
|  | 4b. Negative Aspekte | 4ba. Rollenkonflikte (*n* = 2) durch eingreifendes beziehungsweise dominantes Verhalten  4bb. unzureichende Unterstützung durch fehlende zeitliche Verfügbarkeit genannt (*n* = 2). |
| 5. Koordinationsstelle | N = 5 |  |
|  | 5a. Positives | Alle Therapeut:innen bewerteten die Zusammenarbeit positiv. 5aa. freundliche, einfache und zeitnahe Kontakt (*n* = 3),  5ab. gute Unterstützung zum Beispiel bei der Vernetzung mit anderen Akteuren und rechtlichen Fragen (*n* = 3)  5ac. gute Intervision (*n* = 1). |
|  | 5b. Verbesserungswünsche | 5ba. Screening der Gesundheitspat:innen (*n* = 1),  5bb. Trennung der Rollen Dolmetscher und Gesundheitspate (n = 1) 5bc. mehr sozialarbeiterische Unterstützung der Patient:innen (*n* = 1)  5bd. Anregungen für Fortbildungen zu kultursensitiver Therapie (*n* = 1). |
| 6. Allgemein | N = 14 |  |
|  |  | 13 der 14 Therapeuten (93%) konnten sich vorstellen, in Zukunft Geflüchtete als Patienten zu betreuen. 6aa. Betonung der Wichtigkeit (*n* = 3).  6ab. Machbarkeit ( n= 2)  Bsp: „*Durch das Projekt ist jeder und jede Psychotherapeut/in in der Lage auch den höheren Aufwand etwas abzufedern (durch die Koordinationsstelle) und sprachliche Barrieren und kulturelle Verständnisprobleme zu überwinden (durch Sprachmittler und/oder Gesundheitspaten)“* (PT7). |
